# Supplementary material for: Are carbon emissions trading and green financial instruments synergistic? -Comprehensive quantitative research based on content analysis
Source: PLoS One. 2024 Mar 7;19(3):e0298601. doi: 10.1371/journal.pone.0298601 (PMC10919601; doi:10.1371/journal.pone.0298601)
Supplement: S1 Data — (ZIP) [file pone.0298601.s001.zip › Data/地级市Z_F工作报告.zip!/╝¬┴╓/╦─╞╜╩╨/└┤╫╘╣1⁄2╓┌║┼í╛┬φ┐╦╩2╛▌═°í┐_2010.docx]

各位代表：

　　现在，我代表市人民政府，向大会报告工作，请予审议。

　　一、2009年工作回顾

　　2009年是新世纪以来我市经济社会发展历经考验的一年。面对金融危机等诸多困难和挑战，全市上下团结奋进，迎难而上，积极应对，全力做好保增长、保民生、保稳定各项工作，保持了经济社会平稳较快发展的良好态势。

　　――经济发展快速提升。顶住金融危机对实体经济的冲击，生产总值预计完成690.5亿元，同比增长20.2%，增幅居全省前列，其中，一、二、三产业增加值分别增长5%、33%和17%，三次产业比重调整到26.1：40.6：33.3。全社会固定资产投资预计完成510亿元，同比增长60%，增幅居全省前列。在结构性减税的不利情况下，财政收入保持了两位数增长。全市一般预算全口径财政收入完成43.4亿元，地方级财政收入完成21.4亿元，同比分别增长15.7%和 22.7%。全市财政支出完成90.8亿元，同比增长27%。社会消费品零售总额预计实现234亿元，同比增长17.8%。居民消费价格指数控制在99.5%以下。

　　――项目建设势头强劲。充分利用国家扩大内需和刺激经济增长政策，超前谋划，上争下促，攻坚项目，全市3000万元以上在建项目达到600个，其中亿元以上项目180个，工业项目380个。新建项目用地量同比增长56%，市本级增长121%，出现了四平历史上投资规模大、增长速度快、税源型项目较多的可喜局面。天丰50万吨马铃薯循环利用和30万吨复合肥、金隅100万吨电石渣制水泥、宏宝莱年产10万吨植物蛋白饮料、公主岭吉通电缆和年产2万吨木糖醇等一批重点项目已竣工投产；与西班牙合作的华翔汽车内饰件、巨润生物20万吨大豆蛋白肽、梨树曙光3000万只肉鸡屠宰加工、吉林世宝精密铸件、新加坡唐传科技园10万吨功能糖等一批较大项目扎实推进。能源产业的兴起成为项目建设的一大亮点，伊通莫里青油田开发全年完成投资14.5亿元，新打井105口，建设年处理能力40万吨原油联合站一座；四平电厂二期、双辽电厂二期、华能30万千瓦风电等一批投资超10亿元的大项目将成为经济发展的强力支撑。

　　――工业经济逆势赶超。把决战工业作为经济工作的重中之重，集中精力破解发展难题，实现了速度领先和总量赶超，工业对全市财政的贡献率达到59.6%，成为拉动经济增长的强劲引擎。全市规模以上工业企业达到458户，新增86户。预计实现工业总产值750亿元，工业增加值247亿元，同比增长40.4%和35%，增幅均居全省第一位；工业利润预计实现24亿元，月均增幅保持在50%以上。规模以上工业总产值和工业利润绝对值跃升至全省第四位。十大特色产业园区建设扎实推进，汽车零部件等七个特色产业园区成为省级园区，完成投资45亿元。换热器产业发展规划编制完成，换热器国检中心即将竣工。全市实施重点技改项目104项，预计投资超过100亿元，同比增长33%。申报国家、省级科技项目121项，获省级科技进步奖5项，“超高密度存储材料及器件研究”项目被列入国家“863”计划。全市高新技术企业发展到10户，四平市精细化学品公司被命名为“国家级创新型企业”。四平市生产力促进中心升级为国家级示范中心。节能降耗取得新成效，单位GDP能耗预计下降5.5%。全市工业用电、用水、用气量等支撑工业经济增长的要素增幅居全省前列。全省工业经济工作现场会在我市召开。

　　——发展活力持续增强。政府机构改革顺利完成，机构设置由原来的44个减少到32个，取消行政职能35项。市对区、开发区下放行政审批权限43项。工业企业改制取得积极进展，为220户厂办大集体企业职工发放经济补偿金4.2亿元，31户政策性破产和优化资本结构试点企业13917名退休职工纳入城镇职工基本医疗保险。国有粮食购销企业产权制度改革全面完成，集体林权制度改革稳步推进，市区供水管理体制得到理顺。重新调整市对区、开发区的财政管理体制，市直非税收入管理体制改革取得新进展。金融流动性增强，全市各项贷款余额359亿元，增长15.5%，累计为中小企业新投放贷款近10亿元，增长125%，金融机构贷款增量位居全省第三位；全市新增小额贷款公司9户，担保公司2户；工商银行、建设银行成立了小企业金融服务中心，农行累计发放惠农卡38万张，放款13亿元。我市被评为中国第五批金融生态城市。全面落实国家拉动内需政策，争取国家投资和地方政府债券12.07亿元，争取额度位居全省前列；“家电下乡”销售家电产品6.2万台（部），销售额1.26亿元。统筹编制《四平市旅游发展总体规划》，积极建设旅游精品项目，开展“中国叶赫满族民俗旅游节”等系列活动，全年预计实现旅游收入10亿元。营造良好创业环境，创业指数明显提升，全年预计新增个体工商户5270户，新创办企业487户，民营经济主营业务收入实现780亿元，上缴税金26.4亿元，同比分别增长35.9%、30%，我市被评为全国首批成长型创业之城。以高层次、小分队、专业化招商为主，有针对性地赴长三角、珠三角、环渤海地区进行梯次招商、产业链招商，成功举办了“北京四平同乡联谊会”、“四平市十大特色产业园区推介会”等系列经贸交流活动，全年引进项目897个，实际到位资金351亿元，同比增长50.1%。外贸进出口总额预计完成1.42亿美元，增幅全省第一。巨元换热器公司在美国纳斯达克成功上市，实现上市公司零的突破。开发区、工业集中区快速健康发展，主阵地作用日益凸显，集聚承载能力明显提升。红嘴高新开发区大学科技园被评为省级中小企业孵化基地和大学生创业基地。

　　——农村经济加快发展。战胜严重伏旱，粮食产量达到111.1亿斤，居全省第二位。新建牧业小区379个，肉、蛋、奶产量分别达到115万吨、58万吨和25万吨，牧业产值预计实现220亿元。新建续建水利工程24项，新增农机动力10.5万千瓦，农业生产条件得到改善。全年植树造林15万亩，完成绿化村屯502个，全省“绿化美化村屯、创建绿色家园行动”启动仪式在我市举行。园艺特产业取得新成效，新增棚膜面积8600亩。农民组织化程度不断提升，农民专业合作社发展到1100个，有34个被评为国家和省试点示范社，全省首次农民专业合作社工作会议在我市召开。粮食清仓查库顺利通过国家验收。新农村建设取得新进展，全市四级试点村镇达到384个。全市共有13个小城镇列入全省“百镇建设工程”规划，位列全省第二位，叶赫、孤家子、梨树镇成为全省首批建设镇。全市村村通油路（水泥路）工程建设完成1218公里，行政村通油路（水泥路）达到88.1%。

　　——宜居城市扎实推进。与中国城市规划设计研究院签定了委托编制协议，新一轮城市总体规划修编工作已全面展开。着力把城市资源潜力变为发展优势，建立了城建一级融资平台，首批发行债券申请已报国家发改委待批。城市基础设施建设加快推进，新建、翻建海丰大路、平东大路、新科路等七条主次街路；开工建设河南街、师院西街、南湖西街和市府路；对天桥、南湖街公铁立交桥进行了全面整修；建设平东大路直径1.8米雨水排水管线1717延长米；对东山大路南段两侧人行步道进行了铺装；兴红街公铁立交桥已开工建设，仁兴街公铁立交桥拆迁工作基本结束；完成了紫气大路、东丰路、开运街的土地征用及规划设计工作。全市棚户区改造拆迁面积93.3万平方米，完成施工面积168.1万平方米，市区集中建设廉租房11.8万平方米。城市管网改造力度不断加大，市区新建自来水管线1.64万延长米，新建天然气管线1.2万延长米，新增集中供热能力102万平方米。全市电网建设总投资2.68亿元，增加变电容量196兆伏安，增加线路263公里。全市公路建设完成投资8.01亿元，竣工里程1265公里。生态环境建设取得新进展，市区污水截流干管工程全部完工，污水收集率达到100%。城市生活垃圾处理厂建设完成阶段性目标。加强市区各街路、广场和住宅小区绿化改造，新增城市绿地面积38.3万平方米。勤富水上公园完成征地工作。四叶一级路沿线4个非法采石场被关闭。全市环境质量明显改善，主要污染物化学需氧量和二氧化硫排放量分别削减1200吨和1400吨。切实加强城区环境综合整治，城市管理长效机制进一步完善。市区出租车启用统一号牌。主要街路两侧楼宇及南河河道实现了重点亮化，城市品位大幅提升。

　　——民生工作成果显著。去年初确定的民生实事较好完成。城镇新增就业5.9万人，公益性岗位安置9922人，“零就业家庭”保持动态为零，农村劳动力转移就业64.6万人，城镇登记失业率控制在4%以内。四平籍高校毕业生一周就业做法得到省政府充分肯定。城市居民人均可支配收入15100元，农民人均纯收入5550元，分别增长11％和10％。社会保障进一步加强，养老金和失业金及时足额发放。企业退休人员月基本养老金提高到940元。为16824名职工补发拖欠工资5908万元。为12680名下岗职工全额提取住房公积金2420万元。职工医疗保险及城镇居民医疗保险参保人数分别完成35.4万人和75.9万人。新农合标准提高到每人每年100元，参合率达到80.2%。劳动合同签订率达到96%以上，基本保证了农民工工资及时足额发放。城市最低生活保障标准月人均提高到214元，农村最低生活保障标准年人均提高到760元。妥善处理了棚改9#地遗留问题，3299户动迁户全部回迁。下力量解决供热单位弃管问题，为1180户居民、15.36万平方米住宅按时供热。建设农村饮水安全工程321处，16.32万农民实现了饮水安全。超额完成农村泥草房改造任务，15927户农民喜迁新居。切实保障群众饮食用药安全，食品、药品监管工作经验在全省推广。市儿童福利院投入使用，免费为1082例白内障患者实施了康复手术，社会救助能力进一步增强。

　　——社会事业协调发展。深入开展公民思想道德建设，文明城市创建活动扎实推进。节水型社会建设走在全省前列，全国节水型社会建设中期评估我市获松辽流域第一名。教育事业均衡发展，城市义务教育阶段学杂费全部免除，农村义务教育经费得到保障，全年投入义务教育保障经费9246万元。职业教育快速发展，吉林省农业工程职业技术学院落户四平，四平职大新生录取实现历史性突破，中等职业教育集团化办学经验在全省推广。实施国家“特岗教师”计划，招聘371名高校毕业生充实到农村教师队伍。教师“一评三考”工作被提名为全国首届教育改革创新奖，并作为绩效考核的成功经验在全国推广。全面做好甲流及手足口病防控工作，保障了人民群众生命健康安全。公共卫生服务能力不断增强，中心医院晋升为三级甲等医院，中医医院进行了异地换建改造，妇婴医院新建病房楼投入使用，农村乡镇卫生院改造和城市社区卫生服务中心建设取得新进展，全国民营医院发展经验交流会在我市召开。低生育水平持续稳定，人口计生信息化建设和流动人口计生管理工作走在全省前列，生育关怀工作成为全国典型。扎实推进“文化惠民工程”建设，群众文化活动丰富多彩，成功举办了庆祝建国60周年系列文化活动；积极推进文化产业发展，亿豪动漫产业园落户四平。有线电视网络数字化实现整体平移，全部完成广播电视“村村通”工程。广电大厦主体工程和外装修完工。群众体育蓬勃开展，成功举办了首个“全民健身日”、第五届社区体育大赛等活动。高水平承办了省第五届少数民族传统体育运动会。竞技体育在第十一届全国运动会上取得好成绩。市体育场和全民健身中心主体工程完工。扎实推进国家知识产权试点工作，被评为国家专利执法、专利培训工作先进集体。圆满完成了援建四川黑水县龙坝乡通村水泥路工程。双拥活动深入开展，军政军民关系进一步密切。外事、民族宗教、侨务、人防、地震、气象、邮政、通讯、老龄、地方志等工作取得新的成绩。

　　——行政效能有效提高。坚持依法行政，严格按照法定权限和程序行使权力。自觉接受人大的法律监督、工作监督和政协的民主监督，全年办理人大代表建议96件、政协委员提案189件，办复率达100%。普法工作扎实深入，依法治市工作不断加强。深入开展社会治安综合治理，加大打击刑事、经济、网络犯罪力度，命案破案率达95.2%，圆满完成了国庆安保等工作任务，确保了人民群众生命财产安全和社会安定。认真落实安全生产责任制，严厉查处各类安全生产隐患，扭转了安全生产工作被动局面。全省农村消防工作现场会在我市召开。全面加大信访工作力度，对113个信访疑难案件落实各级政府和部门一把手包案责任制，息访率达到90%以上，解决了困扰多年的站前改造回迁、玻璃制品厂职工债务、疙瘩岭土地纠纷等信访积案,去省、进京非正常访明显减少。深入开展效能监察和审计监督，严肃查处违法违纪案件，经济发展软环境不断改善。建筑面积8100平方米新政务大厅投入使用，所有行政审批项目实现“一站式”办理。严格财税管理，依法规范税费和土地出让金减免缓审批。深入开展学习实践科学发展观活动，政府工作全面加强。

　　这些成绩的取得非常不易，是市委正确领导的结果，是四平广大干部群众攻坚克难、团结奋斗的结果，凝结着全市各个方面的智慧和辛勤汗水。我代表市人民政府，向奋战在全市各条战线的广大劳动者，向给予政府工作大力支持的人大代表、政协委员、各民主党派、工商联、各人民团体和社会各界人士，向关心帮助四平建设的驻平部队、武警官兵、中省直单位及海内外朋友表示崇高的敬意和衷心的感谢！

　　在看到成绩的同时，我们也清醒地看到，我市经济和社会事业发展中还存在一些较突出的问题和差距。主要是，经济总量不大、结构不优，产业综合竞争优势不强，融资瓶颈制约对企业影响较大；项目建设虽实现了历史性突破，但缺少具有战略性、牵动性的大项目；财力增长还不能完全满足社会发展和公共支出需要，收支矛盾非常突出，财政供养人口过于庞大，工资水平还不高；农业基础仍然薄弱，抵御自然灾害能力不足；城市建设和管理还有不尽如人意的地方；由于历史欠账较多，导致一些涉及百姓住房、就业再就业、社会保障等方面民生工程进展较慢，维护社会稳定和化解矛盾的任务繁重；软环境与投资经营者的期望还有较大差距，部分公职人员思想解放程度不够、精神状态不佳、工作作风不实、工作方式陈旧甚至行政不作为。这些问题，必须在今后的工作中认真加以解决。

　　二、2010年工作安排

　　2010年是全面完成“十一五”规划的最后一年，做好今年工作，对我市继续应对金融危机，保持经济快速增长，为“十二五”时期发展奠定良好基础具有重要意义。今年工作总体思路是：坚持以科学发展观统领经济社会发展全局，以党的十七大和十七届三中、四中全会及中央经济工作会议精神为指针，围绕稳增长、转方式、调结构，深入实施工业立市、大上项目、全民招商战略，着力提高城市和企业核心竞争力，着力促进产业集聚培育特色经济，着力推动城乡统筹发展社会和谐进步，着力实施各项民生工程，继续实现“一个巩固、两个领先”，实现四平经济发展新跨越。

　　主要预期目标是：全市完成生产总值810亿元，增长17.3%；一般预算全口径财政收入50亿元，增长15.2%；规模工业增加值284亿元，增长15％；全社会固定资产投资660亿元，增长30%；社会消费品零售总额270亿元，增长15.3 %；城镇居民人均可支配收入实现16610元，农民人均纯收入实现6105元，分别增长10%；居民消费价格指数为103%；城镇登记失业率控制在4.5%以内；人口自然增长率控制在5‰以内；万元GDP能耗下降5%。

　　为实现上述目标，重点做好以下工作：

　　（一）全力推进项目建设，更加注重投资拉动，扩张经济总量

　　攻坚项目促增长。在项目谋划上，紧紧把握国家政策取向、产业导向和投资方向，立足资源优势和四平特色，围绕装备制造业、农副产品加工业、新能源、农业科技示范、现代服务业、医药中间体、节能减排、低碳经济、基础设施、民生工程及文教卫生等经济社会发展重点领域，深入推进项目谋划，加强与知名项目咨询机构合作，谋划大项目、好项目，特别是谋划好投资10亿元以上重大工业项目，市级项目库储备亿元以上项目达到400个以上。在项目摆放上，突出“一核三带”，重点布局四平市区，长平经济带、长郑经济带和长营经济带，培育特色经济，打造产业聚集区。在项目建设上，加速推进湖南中烟四平卷烟厂30万大箱异地改造、双辽电厂二期120万千瓦火电机组、昊华新“双15万吨”离子膜烧碱和聚氯乙烯等一批新项目，促其尽快开工建设。做好大唐公主岭热电联产项目、长春四平双向8车道高速公路项目前期工作。全力推进华能30万千瓦风电、新加坡唐传科技园10万吨功能糖、中国华电那木斯30万千瓦风电、红嘴年产1万辆特种专用汽车、公主岭同心中重卡车桥总成等一批在建项目的建设进度。继续抓好四平热电厂二期60万千瓦发电机组、伊通莫里青石油开发、吉林世宝精密铸件、国能公主岭生物发电、巨润生物大豆蛋白肽、双辽吉利年产300万套轴承改造等一批计划投产项目建设，确保年内投产达效。在项目推进上，充分发挥十个重大项目推进组的作用，继续落实重大项目包保责任制，及时解决项目建设中存在的问题；建立项目建设考核机制，严格考核开工建设、投产达效和企业税收，提高项目管理水平。

　　全面开放促增长。以产业链招商为主攻方向，以东北亚、“环渤海”、港澳台等国家和地区为重点，加大换热器、汽车及配件、农业产业化及食品加工、行走机械、医药等行业专题招商的力度和密度，多引进科技含量高、带动能力强、产业链条长的大项目、好项目。突出南接北融，与长春、沈阳签署区域经济合作协议，积极承接产业辐射和带动，在产业配套、企业合作、科技成果转化、人才交流等方面取得突破性进展，做到错位发展、借力发展。组织高层次、专业化招商小分队，对世界500强及国内百强企业进行叩门招商、跟踪招商。在全国发达城市和有合作关系的国外城市，聘请招商顾问，实行委托招商。全年完成招商引资任务460亿元，同比增长30%。加强资本运作，扩大资金来源，把2010年作为“资本运作年”，推动资本市场融资取得突破。充分挖掘资源、土地、城市等方面的优势，化资源为资本。发展股权投资基金，支持有条件的企业发行企业债券、短期融资券和中期票据；积极引进域外金融机构和创投公司，加强外源性融资；加快信用担保体系建设，集聚民间资本，年内再新增4家担保公司和6家小额贷款公司；鼓励金融机构提供更多灵活的融资业务和金融产品，积极探索应收账款、林权、土地经营权等抵押贷款方式，加大金融支持经济发展的力度，年内设立全市首家村镇银行；制定措施，确保区域内金融机构新吸收存款主要用于当地。对具备上市条件的企业提供上市辅导和政府支持，力争企业上市工作取得新突破。全面落实全民创业各项扶持政策，培育创业主体，孵化创办新企业，年内争取新增个体工商户5000户，新创办小企业500户。全力支持企业扩大出口，优化出口产品结构，全年完成外贸进出口总额1.55亿美元。

　　完善载体促增长。进一步规范开发区、工业集中区管理，改革体制，创新机制，完善功能，优化环境，全力打造发展新优势。招商引资、固定资产投资、工业增加值分别增长40%、40%和30%，建设亿元以上项目25个，5亿元以上项目10个。合理高效利用土地，提高投资强度，开发区达到每公顷3000万元以上，工业集中区达到每公顷2000万元以上。支持开发区、工业集中区融资平台建设，全力破解资金瓶颈。加快基础设施建设步伐，争取完成投资20亿元，增长30%，尽快实现“七通一平”。加快推进开发区、工业集中区进档升级，为承接产业转移、推进产业集聚创造条件。鼓励有条件的开发区、工业集中区建设国家级高新技术重点实验室和工程技术研究中心。

　　（二）全力推进工业立市，更加注重结构优化，提高经济增长质量和效益

　　抓特色，推进产业升级。构建具有四平特色的新型工业体系基本框架，利用3-5年时间，形成区域上相对集中、生产上分工协作、技术上互动创新、资源上互补共享的优势特色产业集群。深入实施工业产业跃升计划，全面推进换热器、汽车零部件、行走机械、农产品及食品加工、玻璃、畜产品加工、光伏、化工、现代服务业、医药中间体10大特色产业园区建设，重点推进换热器和汽车零部件两个产业，用好省扶持换热器特色产业园区发展政策，强力推动企业进园，项目进区，把换热器产业做大做强，力争两年内实现产值100亿元；依托一汽，重点开发汽车内饰、汽车油管总成、卡车车身车架、刮水器、新能源汽车专用零部件等系列产品，打造具有特色竞争优势的汽车零部件产业集群。充分利用我市产业、企业、产品等优势，加强与国内领军企业合作，促成企业重组和产业升级。

　　抓存量，提高质量效益。继续开展服务企业“大篷车”活动，逐户企业解决实际问题，促使企业在应对危机中转型升级。抓好煤电油运等生产要素组织调度，帮助企业争取生产和项目资金，为企业生产经营创造良好条件。推动企业提质增效，用两年时间使50户产业龙头企业实现增量扩能，100户重点企业产值超两亿元，规模以上企业发展到600户以上。加大企业改革力度，按政策为厂办大集体职工接续养老保险关系，年内完成厂办大集体改制任务。加强企业家队伍建设，培养企业领军人物，打造现代企业管理样板企业。推动企业节能减排，重点企业能耗下降10%以上。

　　抓创新，增强工业活力。实施技术改造三年滚动计划，用三年时间实施技改项目300项，完成技改投资300亿元，提高装备和加工制造的整体水平。加速信息技术在企业生产管理环节的应用，强化质量控制。全力实施“五个一批”技术创新工程，搭建合作平台，广泛开展产学研联合、合作，以新材料、新能源和节能环保领域为重点，组织实施一批自主创新技术产业化示范工程项目。培育、发展和创建国家、省级企业技术中心，支持企业建立技术研发机构，确保新产品产值增长20%以上,首次投产新产品达百种以上。建立四平科技创新中心，积极争创“省级可持续发展实验区”。大力实施名牌战略，完善奖励机制，鼓励企业争创中国驰名商标和中国名牌产品，提高四平制造的知名度，巩固“全国质量兴市”先进市成果。

　　（三）全力推进新农村建设，更加注重发展现代农业，促进农村经济全面繁荣

　　坚持用工业化思维谋划农业，以项目建设为统领，加快农业现代化进程。今后一个时期，重点抓好“一个工程、三区、六个基地”，即：增产30亿斤粮食建设工程，20个粮食高产示范区、20万亩棚膜经济区、1000个牧业小区，双1000万头猪繁育饲养基地、10万只梅花鹿养殖基地、50万吨马铃薯生产基地、500万吨玉米深加工基地、1000万头猪深加工基地、5000万只禽屠宰加工基地。加强农业综合生产能力建设，落实惠农政策，保护和调动农民种粮积极性，搞好公主岭省级农业科技示范园区建设，大力实施农业科技集成项目，深入开展粮食高产创建活动；加强农业基础设施建设，全力抓好引松供水、大中型灌区改造、病险水库除险加固、抗旱水源等水利项目，搞好水资源科学调度，抓好黑土区治理工程和农业综合开发项目，加大基本农田保护力度，提高农业机械化水平；扎实推进集体林权制度改革，加强森林资源培育和管护，确保连续30年无重大森林火灾。深入推进“牧业三年攻坚战”，加大牧业小区建设力度，全年新建牧业小区466个，强化动物疫病防控，改良畜禽品种品质，加快建设牧业经济强市。积极发展特色农业，推进园区化、专业化、标准化生产。年内新增棚膜面积9000亩，形成区域特色优势产业带。发展以旅游观光农业、绿色环保农业和精品农业为主的市郊型农业，打造四平特色农业品牌。大力推进农业产业化经营，培育壮大农畜产品加工业，推动重点龙头企业扩规模、上水平、增效益。加快农民专业合作社发展，提升农民专业合作社发展的质量和水平。大力实施“千村提升、百村示范”工程，全面开展农村环境整治攻坚战。加快城镇化步伐，坚持规划先行，强化产业支撑，打造各具特色的工业大镇、旅游强镇、商埠重镇和历史文化名镇，提高城镇综合承载能力。加强农村公益基础设施建设，力争全年建设乡村公路1500公里，实现全市行政村全部通油路（水泥路）。

　　（四）全力推进服务业发展，更加注重发挥区位优势，培育新的经济增长点

　　充分发挥我市区位优越、交通便捷等优势，规划和培育现代物流和连锁配送中心、农机物流中心和农产品物流中心。围绕打造区域商贸中心，重点支持欧亚商贸、国美电器、华生、苏宁、恒客隆等连锁企业加快发展，争取更多的知名品牌进驻四平。加快建设现代服务业特色产业园，大力发展总部经济、服务外包、电子商务、创意产业、投资咨询、广告会展等新兴服务业。加快二手车市场、梨北生猪交易市场、金元蔬菜水果批发市场等建设，增强区域集聚和辐射能力。加快发展农业生产资料经营服务网络、农村日用消费品经营网络、农副产品购销网络、再生资源回收利用网络，健全农村现代流通服务体系。注重消费拉动，继续实施“家电下乡”、汽车以旧换新、购房优惠政策，鼓励住房、汽车等大宗消费，发展社区商业、物业、家政等服务性消费，开发旅游、文化、体育健身等热点消费。认真解决建筑工程领域突出问题，整治房地产市场交易秩序，促进房地产业健康发展。进一步完善旅游总体规划和景点建设规划，挖掘历史文化资源，发展红色旅游、休闲度假游、民俗风情游，打造旅游精品线路，抓好叶赫满族风情旅游带、世界萨满文化生态城等旅游项目建设，继续办好“中国叶赫满族民俗旅游节”等特色旅游节庆活动，扩大四平旅游影响力。着力提升旅游配套服务水平，促进酒店业、茶楼业、餐饮业健康发展，在各县（市）开工建设一批四星级以上宾馆。

　　（五）全力推进宜居城市建设，更加注重提升城市功能，不断改善人居环境

　　高标准完成新一轮城市总体规划和土地利用规划修编。在总体布局上，开发东南、拓展西北，逐步打通环城公路以内的主要规划街路，拓宽城市发展空间。继续抓好“蓝天、碧水、宁静、亮化、洁净、畅通、生态、安全”等八大工程。重点抓好城市建设六件实事：一是新建紫气大路、东丰路、开运街，翻建四经街、邮电街等主次街路。二是开工扩建南湖街公铁立交桥，实现仁兴街、兴红街两座公铁立交桥建成通车。三是全市完成棚户区改造拆迁面积50万平方米，建设面积80万平方米。四是提高城市亮化水平，对市区现有路灯进行LED节能改造，完成所有次干路路灯照明工程。统一设计、动静结合，对市区主要街路两侧楼宇实施高档次亮化。五是不断提高国家园林城市绿化水平，加快建设勤富水上公园，在城区西部和南部谋划建设100-200米宽的森林公园。六是力争用三年时间，在紫气大路两侧，建起一座近6平方公里，集商业、居住、休闲、娱乐为一体，贴近自然的生态新城。提升国家卫生城市水平，深入开展城区环境综合整治，彻底清理城区垃圾，加大拆除违法建筑力度，严加整治非法营运出租车、三轮车，加强犬类管理。加大资金投入，积极发展城市公交。切实加强城市地下人防工程的开发利用。围绕经营城市，在城市建设融资上实现新突破，鼓励各类投资主体参与市政公用设施建设运营。加强土地集中统一管理，完善土地收储运作机制，严格土地出让金收支管理。

　　（六）全力推进社会事业，更加注重改善民生，促进社会和谐进步

　　促进城乡义务教育均衡发展，完善义务教育经费保障机制，大力发展职业教育，重视学前教育和特殊教育，加强教师队伍建设，提高教育质量。加快中小学校舍安全工程建设，年内完成全市90%校舍维修和重建任务。推进医药卫生体制改革，完善公共卫生服务体系。完善农村三级卫生服务网络，加快县级医院、乡镇中心卫生院改造，提高农村医疗卫生服务水平。深化文化体制改革，繁荣文化事业，发展具有四平特色的文化产业。全面完成广电大厦建设，完善广播影视系统基础设施，提高广播电视节目的制作和播出质量。综合治理人口计生工作，继续深入开展生育关怀活动，稳定低生育水平。加快发展邮政、通讯事业，更好地服务经济社会发展。积极备战和参加省第十六届运动会，力争取得好成绩。大力推进体育设施建设，确保市体育场和全民健身中心投入使用。

　　把就业作为民生之本，着重解决大学生、农村劳动力和城镇就业困难人员就业问题，加强职业培训，落实各项扶持政策，全年城镇新增就业5.05万人，公益性岗位安置9700人，农村劳动力转移就业52.8万人。把保障作为民生之基，全年职工医疗保险、居民医疗保险、工伤保险、生育保险参保人数达到36万人、77万人、17万人和19.5万人。积极开展养老保险的流转、接续和新农保试点工作。继续发展新型农村合作医疗，提高筹资标准和参合率。全力做好甲流等传染性疾病防控工作，切实减轻疫情危害。加大各级财政投入，实现城乡居民最低生活保障全覆盖。积极推进城市廉租房建设，解决中低收入家庭的居住困难问题。加大城乡医疗救助力度，提高灾害应急能力和救助水平。关心支持国防和军队建设，扎实做好双拥工作，为争创全国双拥模范城“六连冠”打好基础。高度重视民族宗教工作，加强宗教事业管理，维护民族团结局面。搞好残疾人职业技能培训，开发残疾人就业岗位，建设多功能“残疾人康复服务大楼”。深化城市供水体制改革，推进水务一体化。完成农村饮水安全工程、农村困难户泥草房改造任务。解决好“暖房子”问题，加快热源能力建设，加强供热监管，有效保障城市冬季供热。

　　完善矛盾纠纷排查化解机制，继续落实信访包案制，集中力量解决涉及民生的信访问题，妥善处置社会矛盾和群体性事件，提高超前性、及时性、实效性，把问题解决在萌芽之中。不断深化法治宣传教育，加强社会治安综合治理，继续保持对刑事犯罪的高压严打态势，增强人民群众的安全感。突出预防为主，抓好重点行业和重点部位公共安全专项整治，加大城市视频报警及监控系统建设力度，安装探头突破1万个点。全面落实各级政府监管责任，加大安全生产投入，确保不发生重特大安全生产事故。整顿和规范市场秩序，严厉打击传销等违法行为。加强食品药品监管，建立食品安全综合协调机制，保障人民群众身体健康和生命安全。

　　今年要开展“十二五”规划编制工作。要准确认识经济社会发展趋势和市情，围绕调整结构、转变发展方式、改革开放和改善民生等重大问题，加强战略研究，在更高起点上谋划制定四平未来发展蓝图。

　　（七）全面加强自身建设，更加注重提高执行力，建设人民满意政府

　　建设务实政府。解放思想，真抓实干，抢抓机遇，破解难题，一心一意谋发展，全心全意惠民生，尽心竭力保稳定。少说多做，重实干不张扬，建设一支说话实、干活猛、作风硬、推进工作力度大的公务员队伍，敢于负责，勇于担当，戮力同心，把四平的事业搞上去。坚决纠正疏于干事、互相推诿的不良风气,强化部门之间协调配合，做到政令畅通，指哪打哪。体察民情，厚待百姓，多办利民生、顺民意的实事好事。对机关干部严管厚爱，逐步落实津贴补贴，提高生活待遇。

　　营造良好环境。推进服务型政府建设，牢固树立大局意识、发展意识，真心实意为企业、为基层、为群众排忧解难。简化工作程序，提高办事效率，该办的事情马上办、主动办、上门办，能办的事情工地办、车间办、现场办。以打造投资成本最低、服务环境最好和创业发展高地为目标，为投资者和创业者提供亲情化、保姆式、全天候服务，快查严处涉软案件，着力营造亲商、安商、富商的社会氛围。

　　坚持依法行政。规范行政行为，减少随意性，提高公信力。按客观规律办事，完善公众参与、专家论证的科学决策机制，实行“阳光政务”。自觉接受人大法律监督、工作监督和政协民主监督，接受社会监督和新闻舆论监督。加强行政执法规范化建设，严格按照法定的权限和程序履行职责，公正执法。

　　强化从严治政。全面落实党风廉政建设责任制，扎实推进惩治和预防腐败体系建设，从源头上预防和惩治腐败。加强重点领域、重点工程、专项资金的财政监督、审计监督和廉政监察，严惩商业贿赂行为。以解决群众反映的突出问题为重点，坚决纠正行业不正之风，严肃查处各种违法违纪行为。继续保持艰苦奋斗的优良传统，做到清正廉洁，勤俭办事业。

　　各位代表，四平的发展正处在夯实基础、积蓄能量、加快复兴的关键时期。立足新起点，应对新挑战，实现新跨越，是时代赋予我们的光荣使命。让我们在市委的领导下，紧紧团结和依靠全市各族人民，振奋精神，克服困难，顽强拼搏，扎实工作，为实现四平振兴、人民富裕的目标而努力奋斗！
